# National Surveillance of Injury in the Republic of Korea: Increased Injury Vulnerability in the Late Middle Age

**DOI:** 10.3390/ijerph18031210

**Published:** 2021-01-29

**Authors:** Hansol Chang, Ji Young Min, Dajeong Yoo, Se Uk Lee, Sung Yeon Hwang, Hee Yoon, Won Chul Cha, Tae Gun Shin, Ik Joon Jo, Taerim Kim

**Affiliations:** 1 Samsung Medical Center, Department of Emergency Medicine, Sungkyunkwan University School of Medicine, Seoul 06351, Korea; briquet90@naver.com (H.C.); seukemmd@gmail.com (S.U.L.); gerup@hanmail.net (S.Y.H.); wildhee@gmail.com (H.Y.); docchaster@gmail.com (W.C.C.); tackles@naver.com (T.G.S.); Ikjoonjo@smasung.com (I.J.J.); 2 Department of Digital Health, Samsung Advanced Institute for Health Science and Technology (SAIHST), Sungkyunkwan University, Seoul 06355, Korea; wnet50094@gmail.com; 3 Samsung Medical Center, Research Institute of Future Medicine, Seoul 06351, Korea; oaoa93@naver.com; 4 Health Information and Strategy Center, Samsung Medical Center, Seoul 06351, Korea

**Keywords:** wounds and injuries, epidemiology, age factors, age groups

## Abstract

Surveillance of injury patterns and comparisons among different age groups help develop a better understanding of recent injury trends and early prevention. This study conducted a national surveillance of injury by age group. Data were collected retrospectively from Emergency Department-Based Injury In-Depth Surveillance (EDIIS) in South Korea, between January 2011 and December 2017. Patients were divided into the following four groups by age: Group 1–18 to 34 years, Group 2–35 to 49 years, Group 3–50 to 64 years, and Group 4—≥65 years. A total of 1,221,746 patients were included in the study. Findings revealed that, each year, the injury rate increased in the population aged ≥65 years. The place and mechanism of injury in Group 3 were similar to those in younger age groups, while injury outcomes and injured body parts were similar to those in Group 4. Further, hospital admission rate, ICU admission rate, hospital death, traumatic brain injury, and injury severity increased with an increase in age. In our study, each age group showed diverse characteristics pertaining to the mechanism, place, time, and outcomes of injuries. Interestingly, Group 3, which represented the late middle age, exhibited increased vulnerability to injury, and emerged as a gray zone between the young and old age groups. Therefore, different injury prevention methods are needed for each age group. Specifically, early prevention methods need to be implemented from the late middle age to improve the old age group’s injury outcomes.

## 1. Introduction

Injuries lead to severe health loss, including both mortality and moderate to severe disability across all ages [1]. According to the Global Burden of Disease (GBD) data, in 2017, 4.2 million individuals died by injury, which comprised 8% of the total global deaths [2,3]. The Disability-Adjusted Life Years (DALY) for injury, which is the number of years lost due to ill-health, disability, or early death, is 252 million years, representing 10% of the DALY for more than 100 diseases in 2017 [2,4]. Considering that 4.2 million people died by injury in 1990, which was 9% of the total global deaths, even after two decades, the rate of death from injury has not improved significantly, and it continues to be the main cause of health loss [2].

Since 1940, the view of injuries as “trauma” or “accidents”, resulting in the neglect of the need for prevention, has been changed to that of focusing on prevention [5,6]. Surveillance systems such as GBD and those on injuries, risk factors identified by World Health Organization (WHO), and the Web-Based Injury Statistics Query and Reporting System developed by the Centers of Disease Control and Prevention in the United States consistently provide comparative descriptions of injury [7]. These data and surveillance systems can lead to new insights on injury patterns, aiding the identification of at-risk populations and finally addressing intervention policy decisions [7]. Therefore, various strategies to prevent injuries, such as increasing helmet use among motorcycle drivers, and enforcement of laws pertaining to drunk driving and seatbelt use, have been implemented worldwide [7,8].

The risk of injuries in a population may stem from a wide range of combinations of intrinsic and extrinsic risk factors [9,10,11]. Several studies have examined the spectrum of risk factors for injury in specific age groups, indicating that while injuries in young adults may be affected by alcohol intoxication and risk-taking behaviors, those in older adults may be related to comorbid conditions and reduced motor function [12,13,14,15,16]. Some studies have reported differences in the mechanism of injury across age groups, which is only a part of injury characteristics [12,15]. The mechanism is only a part of the characteristics of the injury and should be considered for the analysis of the injury along with its other influencing (or risk) factors, such as the environmental factors of place, time, and policies, and the host factors of age, sex, and safety gear of the patient [17].

Globally, the population structure has been changing over the years [3,18]. The proportion of individuals at the old age is increasing [16,19,20]. Considering that many injuries are preventable, those occurring in any population can led to lifelong disability that necessitates critical and rehabilitative care, and could increase mortality [1,17,21]. Former surveillance shows that different age groups exhibit different injury mechanisms and outcomes [10,11,13]. Therefore, surveillance of injury patterns and comparison among different age groups are required for a better understanding of recent injury trends and early prevention.

The present study aimed to conduct national surveillance of injury by age group. We intended to investigate intrinsic and extrinsic risk factors, especially by age group, mechanism of injury, place of injury, time of injury, transport injury event patterns, injured body parts, and outcome differences to identify new strategies for injury prevention in the targeted population.

## 2. Materials and Methods

### 2.1. Study Design and Data Collection

Data were collected retrospectively from the Emergency Department-Based Injury In-Depth Surveillance (EDIIS) in South Korea. The study included patients’ data between January 2011 and December 2017. The Korean Statistical Information Service database was used to analyze the total population aged over 18 years to compare it with the number of injured patients aged over 18 years [22]. Accordingly, patients aged over 18 years were included in the study. Specifically, patients were divided into the following four groups by age: Group 1, 18–34 years; Group 2, 35–49 years; Group 3, 50–64 years; and Group 4, ≥65 years [16,23,24].

The EDIIS is based on the dataset of the International Classification of External Causes of Injuries (ICECI) managed by WHO. This database includes the patients’ prehospital records, clinical findings, International Classification of Disease 10th Revision (ICD-10)-based diagnosis, treatment, dispositions in the Emergency Department (ED), inpatient information, demographics, and injury-related factors. The database initially compiled data from 20 EDs in Korea, and a total of 25 EDs participated from 2015.

The EDIIS is a high-quality injury database based on data from EDs in Korea. Physicians who have been introduced to the EDIIS obtain medical information from injured patients. Each ED has coordinators for collecting standard data, and the Korea Centers for Disease Control (KCDC) regularly monitors the quality of the data.

This study was approved by the Institutional Review Board (IRB) of Samsung Medical Center, IRB No. 2020-05-042. The need for informed consent was waived because of the retrospective, observational, and anonymous nature of the study.

### 2.2. Measure

Participants’ age and gender were recorded and they were grouped into four categories by age, as mentioned above. We classified the time of injury as day (07:00 to 14:59), evening (15:00 to 22:59), and night (23:00 to 06:59) according to the 8 h hospital shift, and days of injury as weekdays from Monday to Friday, and as the weekend from Saturday to Sunday [25].

We investigated the injury mechanism and place by group. We modified the mechanism of injury in the EDIIS data based on the ICECI (Table S1). The following mechanisms of injuries were included: transport injury event, falling, slipping, blunt force, piercing/penetrating force, other mechanical force, physical-over-exertion, exposure to chemical or other substances, thermal mechanism, threat to breathing, exposure to natural disaster or other forces of nature, and others and unknown. Fall and slip-down injuries were analyzed separately from blunt injury. The transport injury event was separated from the blunt force injury category.

The activity that the patient engaged in when the injury occurred was categorized based on the EDIIS and ICECI, as follows: vital activity, leisure, play or travel, paid work, unpaid work, sports and exercise during leisure time, and others and unknown.

The place of occurrence was examined based on the EDIIS, and it was reclassified as transport area; home; commercial, recreational, and cultural area or public building; sports and athletics area; farm, industrial or construction area; countryside; medical service area; school/educational area; residential institution; and others and unknown.

Since 2016, data on safety gear have been collected. In case of a transport injury event, we also investigated whether the patient was wearing a seatbelt, or whether an air bag was included and activated when the injury occurred in a car.

The injured anatomical sites were distinguished using relevant ICD-10 codes [26]. Specifically, S00 to S09: Head injury, S10 to S19: Neck injury, S40 to S39: Shoulder and upper-arm injury, S50 to S59: Elbows and lower-arm injury, S60 to S69: Wrists and hands injury, S70 to S79: Hip and thigh injury, S80 to S89: Knees and lower-leg injury, S90 to S99: Ankles and feet injury, S20 to S29: Thorax injury, and S30 to S39: Abdomen and pelvis injury, including lower back and genitals. We surveyed the patients’ injury site using their diagnostic ICD-10 code.

### 2.3. Outcomes

Injury outcomes were assessed based on the length of hospital stay, general ward admission, ICU admission, in-hospital mortality, ED death, injury severity, and traumatic brain injury (TBI). The presence of concomitant injuries was also included.

In this study, we used the Excess Mortality Ratio-adjusted Injury Severity Scale (EMR-ISS) to classify injury severity. The EMR-ISS is a validated tool/method to measure injury severity using the International Statistical Classification of Diseases and Related Health Problems 10th Revision, Clinical Modification (ICD-10-CM) [18]. The EMR-ISS score is classified into the following four levels of injury severity: Mild (1–9), moderate (9–24), severe (25–74), and critical (≥75 or death). For the logistic regression analysis, we divided the EMR-ISS score into two groups: mild and moderate (≤25) versus severe and critical (≥25).

We also recorded whether TBI was one of the severe clinical outcomes. TBI was defined by ICD codes F07.2, S02.0, S02.1, S02.3, S02.7, S02.8, S02.9, S06, S07.1, T90.2, and T90.5.

### 2.4. Statistical Analysis

Continuous variables were described as medians with Interquartile Ranges (IQRs), and categorical variables were described as frequencies with percentages. We used the Kruskal–Wallis test for continuous values and the chi-square test for categorical values to conduct comparisons among age groups. To evaluate the association between injury-related variables (age group, sex, mode of arrival, day of injury, time of injury, day of ER visit, time of ER visit, place, insurance, mechanism, activity, alcohol use, and intent) and outcomes (EMR-ISS, TBI, and mortality), we conducted a univariable and multivariable logistic regression. *p* < 0.05 was considered statistically significant for all statistical tests. R version 4.0.2 was used for the statistical analysis.

## 3. Results

### 3.1. Population

**Figure 1:**
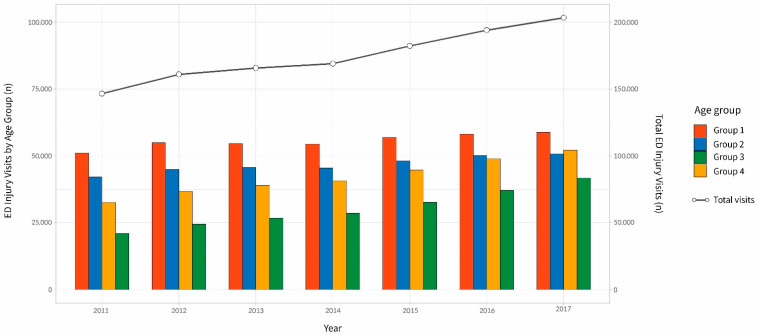
Emergency Department (ED) visits for injury by age group and total ED visits during 2011–2017. Group 1 (18–34 years old). Group 2 (35–49 years old). Group 3 (50–64 years old). Group 4 (65 years and over).

A total of 1,221,746 patients were included in our study. The age groups (Group 1, 2, 3, and 4) comprised 388,622, 326,790, 293,397, and 211,837 patients, respectively (Figure 1).

**Figure 2:**
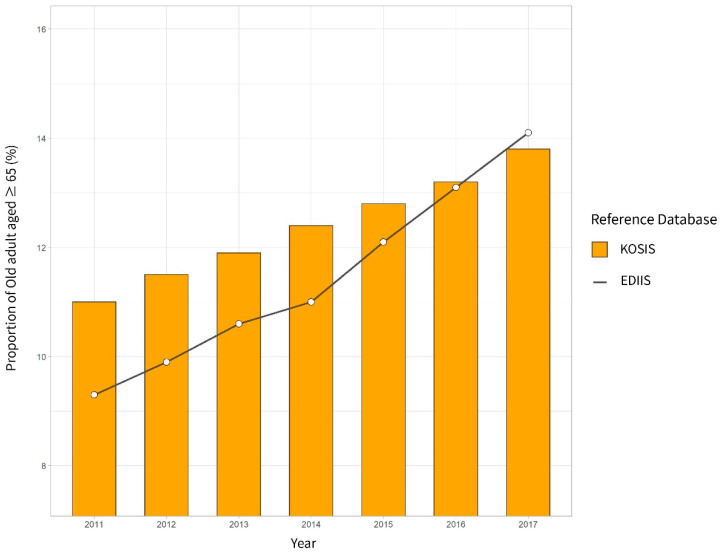
Proportion of older adults aged ≥65 years computed using the Emergency Department Based Injury In-Depth Surveillance (EDIIS) for patients with injury and the Korean Statistical Information Service (KOSIS) for the total population.

Each year, the injury rate increased in the population aged ≥65 years (2011—9.30%, 2012—9.89%, 2013—10.61%, 2014—10.98%, 2015—12.08%, 2016—13.06%, and 2017—14.13%). This increase was even higher than that observed in the national population of adults aged ≥65 years documented in the KOSIS (2011—11.0%, 2012—11.5%, 2013—11.9%, 2014—12.4%, 2015—12.8%, 2016—13.2%, and 2017—14.3%) (Figure 2) [22].

### 3.2. Demographics

Table 1 presents the demographics, pre-hospital information, and general characteristics of injured patients by age group. The proportion of male patients was 62.2% in Group 2, 61.1% in Group 1, 59.3% in Group 3, and 46.2% in Group 4. The use of emergency medical services (by calling 119) decreased with age (36.8%, 29.1%, 24.6%, and 20.3% in Group 4, 3, 2, and 1, respectively).

### 3.3. Time and Place

Across all groups, the number of injuries was higher in the evening (15:00 to 22:59, total *n* = 547,591) than during the daytime (07:00 to 14:59 pm, total *n* = 376,273). Individuals in Group 4 (*n* = 92,613, 43.7% of Group 4) experienced more injuries during the daytime, while those in Group 1 (*n* = 129,165, 33.3% of Group 1) experienced more injuries in the night (0:00 am to 8:00 am, total *n* = 297,868) (Table 1).

**Figure 3:**
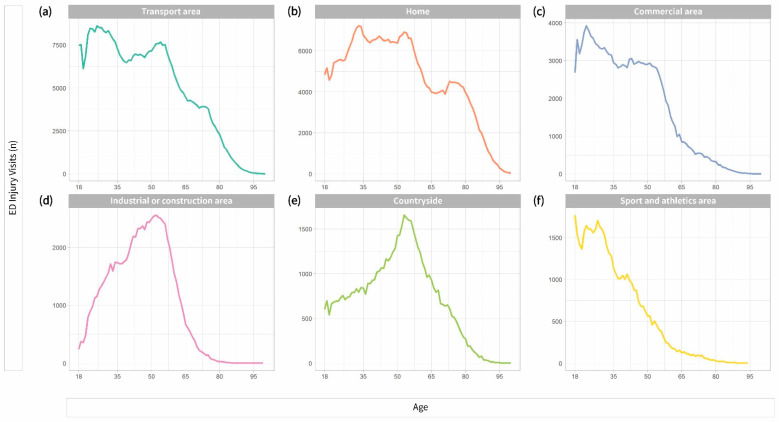
Six of the most common places of injury by age. (**a**) Number of injuries in the transport area (including roads) by age; (**b**) number of injuries at home by age; (**c**) number of injuries in commercial area by age; (**d**) number of injuries in industrial or construction facilities by age; (**e**) number of injuries occurring countryside (including sea and river) by age; (**f**) number of injuries in sport and athletics area by age. ED: Emergency department.

**Figure 4:**
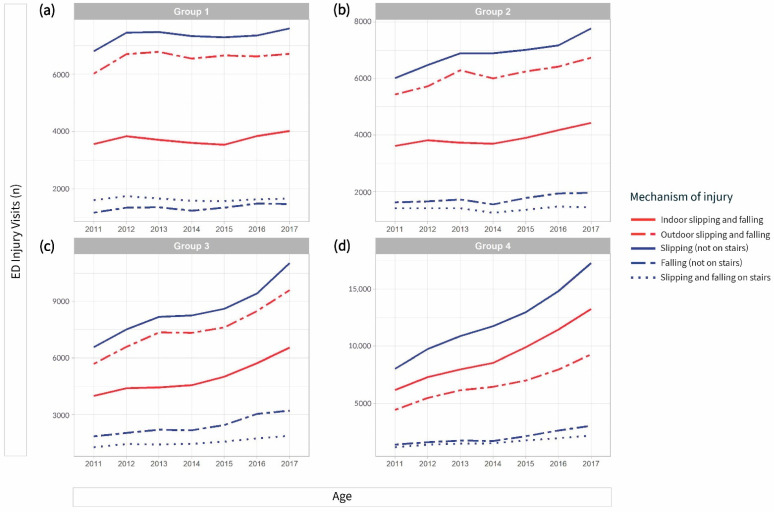
Subgroup analysis of slipping and falling in the injury mechanism by age group. (**a**) Group 1 (18–34 years old), (**b**) Group 2 (35–49 years old), (**c**) Group 3 (50–64 years old), (**d**) Group 4 (≥65 years). The red line indicates a subgroup that was divided into indoor and outdoor. The blue line indicates another subgroup divided into slipping and falling, which did not occur on stairs or occurred on stairs.

The transport area was the most common place of injury in most groups—33.7% in Group 1, 31.8% in Group 3, and 30.1% in Group 2. (Table 1). Those in Group 4 exhibited a higher occurrence of injuries at home or at medical service area. In contrast, those in Group 1 and 2 frequently experienced injuries outdoors, such as at the countryside, sea, river, and transport area, or at commercial, recreational, and cultural areas or public buildings. Group 2 and 3 experienced more injuries at the industrial, or construction area. With an increase in age, the difference between the rate of outdoor and indoor injuries decreased (Figure 3). The subgroup analysis on falling and slipping in terms of the injury mechanism, Group 4 was the only group that exhibited indoor injuries more frequently as compared to outdoor injuries (Figure 4).

### 3.4. Injury Mechanism and Activity Engaged in during Injury Occurrence

Transport injury events, falling, slipping, blunt force, piercing, and penetrating force were the most common injury mechanisms, comprising about 80% of the injury mechanisms in all age groups. Falling and slipping were the most common injury mechanisms in Group 4 (*n* = 112,310, 53.0% of Group 4) and Group 3 (*n* = 88,299, 30.0% of Group 3) (Table 2). Blunt force injury was most common in Group 1 (*n* = 94,262, 24.3% of Group 1) and Group 2 (*n* = 71,339, 21.8% of Group 2) (Table 2).

Across all age groups, vital activity was the most common activity in which the patient engaged during injury occurrence. The rate of injury during leisure, play, or travel was the highest in Group 1 (*n* = 83,780, 21.6% of Group 1), while that during paid work was the highest in Group 3 (*n* = 54,781, 18.6% of group 3).

### 3.5. Transport Injury Event and Safety Gear

The rate of occurrence of transport injury events while in a car is presented in Table 3. The study also investigated whether safety gear was included or activated during transport injury events in a car. In Group 4, 33.1% of the injured patients were reported to not have worn a seat belt when the injury occurred, while the same was 26.6% in Group 1. This rate was higher than that observed in Group 3 (22.1%) and Group 2 (18.8%). In addition, only 27.3% and 23.9% of those in Group 3 and 4, respectively, had air bags in their cars (Table 3).

### 3.6. Injured Body Parts

**Figure 5:**
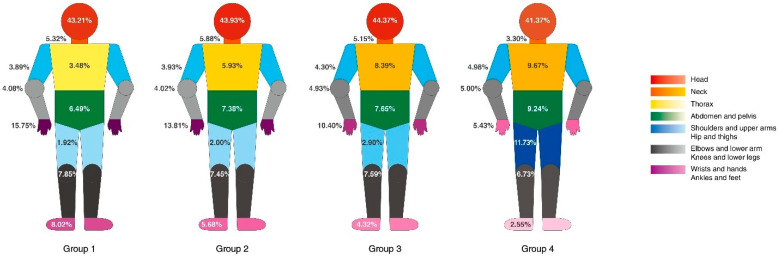
Anatomical sites of the injury according to age group analyzed by International Classification of Disease 10th Revision (ICD-10) codes. Group 1 (18–34 years old), Group 2 (35–49 years old), Group 3 (50–64 years old), Group 4 (≥65 years), presented respectively from left to right). Body parts are indicated by color. Higher color saturation indicates a higher injury rate.

The head was the most commonly injured body part across age groups—43.21%, 43.94%, 44.37%, and 41.37% in Group 1, 2, 3, and 4, respectively. With an increase in age, the rate of injury increased in the following body parts (in parentheses, percentages have been reported for Group 1, 2, 3, and 4, respectively): Thorax (3.4%, 5.93%, 8.30%, 9.67%), abdomen and pelvis (6.49%, 7.38%, 7.55%, 9.24%), shoulder and upper arms (3.89%, 3.93%, 4.30%, 4.98%), and hip and thigh area (1.92%, 2.00%, 2.90%, 11.73%); while it decreased with age in the following body parts: Wrist and hands (15.75%, 13.81%, 10.40%, 5.43%) and ankles and feet (8.02%, 5.68%, 4.32%, 2.55%) (Figure 5).

### 3.7. Outcomes

The hospital admission rate, ICU admission rate, hospital death, traumatic brain injury, and EMR-ISS score increased with age, as did the chances of multiple injuries. The percentage of patients who experienced injuries in more than one body part in the same group was 13.9% (*n* = 29,355) in Group 4, 12.0% (*n* = 35,315) in Group 3, 10.1% (*n* = 33,050) in Group 2, and 9.6% (*n* = 37,245) in Group 1 (Table 4).

We utilized univariable and multivariable logistic analysis identify statistical differences in outcomes between age groups (EMR-ISS, hospital death, and TBI). Findings revealed that age-group based differences were statistically significant (*p* < 0.001) for EMR-ISS, hospital death, and TBI in the multivariable regression. With Group 1 as the reference, the OR for the EMR-ISS score was 1.19 (confidence interval [CI] = 1.17–1.22) in Group 2, 1.61 (CI = 1.58–1.65) in Group 3, and 2.29 (CI = 2.24–2.34) in Group 4. The odds ratio (*p* < 0.001) for hospital death with Group 1 as the reference was 1.56 (CI = 1.46–1.66) in Group 2, 2.4 (CI = 2.25–2.55) in Group 3, and 4.68 (CI = 4.4–4.97) in Group 4. The odds ratio (*p* < 0.001) for TBI with Group 1 as the reference was 1.11 (CI = 1.09–1.13) in Group 2, 1.42 (1.39–1.44) in Group 3, and 1.6 (1.57–1.63) in Group 4 (Table 5).

## 4. Discussion

This is the most recent nationwide study that revealed various injury patterns in different age groups. As this was a multicenter study, the characteristics, place, and severity of injuries were also examined for each age group. We observed that each age group had a different injury pattern, thus necessitating the utilization of different approaches and interventions based on age group. In addition, as the analyses were conducted for each age group, prevention methods can be established in the early stages by considering both present and future age groups. For example, attempts can be made to prevent injury and disability at the old age by preventing the same during the middle age.

Group 1, which represented the young age group, exhibited more injuries in commercial, recreational, cultural areas or public buildings, and in sports and athletics areas. Evidently, their injuries are related to leisure activities. Additionally, they exhibited more injuries in the upper and lower extremities than in the body’s core or trunk. Therefore, injury prevention in this group should be related to outdoor leisure spaces. Since young adults tend to use safety gear less often as compared to other age groups, which is similar to the result of a previous study it is essential to provide safety education for this age group [27]. It is important to target and prevent injury and disability among young adults because they would have more long-lasting effects, and would involve greater socioeconomic costs [9,14].

Group 2, which represented the early middle age group, had similar injury characteristics to Group 1. During this age (35 to 50 years), individuals generally tend to engage in economic activities. Our study also showed that work-related injuries increased from this age. Some previous studies and our study showed that occupational injury is common at this age; therefore, workspace safety needs to be emphasized [28,29]. This group also exhibited a higher incidence of injuries in commercial, recreational, cultural, or public buildings.

Group 3 (50 to 65 years), which represented the late middle age group, also exhibited similar place and injury mechanisms to Group 1. In addition, because this group continues to engage in economic activities, they too exhibit more frequent workplace injuries. to However, the injury outcomes and injured body parts of this group were similar to those of Group 4. The rate of injuries occurring during leisure, pay, or travel activities increased in Group 3 as compared to that observed in Group 2. In addition, the rate of falling injury increased as compared to that observed in Group 1 and 2. The occurrence of daytime injuries also increased, and so did the occurrence of TBI, death, and admission rate. These findings may suggest that injuries occurring in the middle age could be a starting point for later injury and disability. According to the Korea Static Annual Report (2018), individuals in their fifties comprise the largest proportion of injury patients [30]. This suggests that the implementation of old-age injury prevention strategies needs to begin from the middle age.

Group 4 represented the old age population. This group exhibited frequently occurring indoor injuries and most severe outcomes across all age groups. Injury in old age may lead to severe outcomes due to age characteristics, underlying diseases, and medications that compromise vital sign monitoring or coagulation [16]. Falling and slipping were the most common injury mechanisms in this group, which has already been reported in previous studies [16,31,32]. Further, it was observed that this group exhibited more frequent injuries to their core body parts like the trunk or hip as compared to the young age group [33] Indeed, such injuries are more likely to result in damages to vital organs or ambulation disabilities. These findings related to the old age group suggest that injury prevention strategies for this age group need to focus on indoor places, medical facilities, and housing facilities. Additionally, families with older adults are recommended to install home safety devices to avoid falls.

The number of older individuals aged ≥65 years has been increasing globally [4,34]. According to the American National Trauma Data Bank, the proportion of this population ≥65 years in Level 1 and 2 trauma centers increased from 23% in 2003 to 30% in 2009 [31]. In 2010, the rate of population increases in this age group ≥65 years was higher than that of the entire population [19]. Older adults often tend to have a poorer physical function than those in other age groups [35]. However, they do not consider themselves as being aged, and therefore, do not acknowledge their risk of injury [36]. The increased injury rate among older adults might be associated with their maintaining the same lifestyle as they had when they were young, despite the deterioration in their physical functions [37]. Our study revealed that the injury rate in this age group was higher than the rate of population increases among older individuals. Therefore, there is a higher chance of an increase in old-age injury [38]. As mentioned above, the present study found that injury outcomes increasingly worsened from Group 3, the middle-aged group, even though their places or mechanisms of injury were similar to younger groups. Therefore, the middle age can be a starting point for disability and injury prevention. Education about injury prevention and behavior modification in this age could help prevent injuries with severe outcomes at a later age (i.e., in Group 4).

Interestingly, in our study, Group 4 and Group 1 were unlikely to have worn a seatbelt when a transport injury event occurred in a car. Middle-aged individuals (Groups 2 and 3) were more likely to use a seatbelt. This is a remarkable finding because studies conducted in other countries reported that the older age group was more likely to wear a seat belt [39,40]. According to the Korean National Police Agency, the most common cause of traffic accidents was “not performing safe driving” [41]. Older individuals may not be aware of safety devices because Korea does not require another test for the renewal of driving licenses. As a safety belt has already been proven to improve survival in transport injury events in cars, especially among older individuals, implementing mandatory traffic safety education for this population could help reduce injuries [7,42,43].

To prescribe proper medication and vaccination, it is important to know about a disease. Similarly, to devise appropriate injury prevention strategies, it is essential to know the characteristics of injury. Since our study revealed the injury characteristics of different age groups, it may be possible to establish different age-appropriate methods to prevent them. For example, referring to the result of our study, Group 1 will need to be prevented from outdoor injuries via education or the use of proper safety gear for specific activities. For Group 2, imperative policies and regulations to reduce occupational injury will be needed. Group 3 will need education programs for understanding physiological changes due to aging and age-appropriate activities. As injuries among Group 4 participants occurred frequently in housing places, setting up safety gear in-home and in housing facilities will be required. Further research is required to establish targeted and specified injury prevention methods.

### Limitation

This study has some limitations. First, it only included patients who visited the ED, which might have caused a selection bias. Patients with mild injuries who visit other clinics, or those who died on the scene were not included in this study. Second, this study was based on the EDIIS, and the number of EDs listed in this database changed in 2015. Specifically, three EDs were added to the database from 2015 to 2017. Third, we used the EMR-ISS instead of the ICISS to determine injury severity in this study. The EMR-ISS is not used commonly in other countries [18]. However, in Korea, it is used extensively, and several studies have been published based on the same [12,15,44]. Fourth, this was a retrospective observational study based on EDIIS data. These data may have been compromised during coding. However, coordinators at EDs continually monitor the quality indicators of these data and make appropriate corrections. In addition, this is a multicentered database, and the sample is large enough to overcome this limitation. Fifth, before 2015, data regarding whether the patient underwent surgery was not included.

## 5. Conclusions

In our study, each age group showed diverse characteristics in the injury mechanism, place, time, and outcome, and the late middle age group emerged as a gray zone between the young and old age groups. Therefore, it is imperative that different injury prevention methods are developed for each age group; this would include the provision of safety gear in young age, especially for outdoor activity, focusing on occupational injury in early middle age, and preventing falls in housing facilities in old age. Specifically, early prevention methods need to be implemented from the late middle age to improve injury outcomes in the old age group.

## Figures and Tables

**Table 1:** General characteristics of injury patients by age group.

Age Group (Years)	Group 1 (18–34) (*n* = 388,622)	Group 2 (35–49) (*n* = 326,790)	Group 3 (50–64) (*n* = 294,497)	Group 4 (≥65 Years) (*n* = 211,837)	*p* Value
Sex					<0.001
Male	237,639 (61.1%)	203,357 (62.2%)	174,565 (59.3%)	97,974 (46.2%)	
Female	150,983 (38.9%)	123,433 (37.8%)	119,932 (40.7%)	113,863 (53.8%)	
Mode of arrival					<0.001
Walk-in	293,525 (75.5%)	227,230 (69.5%)	183,158 (62.2%)	99,568 (47.0%)	
EMS (119)	78,804 (20.3%)	80,387 (24.6%)	85,781 (29.1%)	77,946 (36.8%)	
Private ambulance	14,608 (3.8%)	17,601 (5.4%)	24,012 (8.2%)	33,305 (15.7%)	
Others and unknown	1685 (0.4%)	1572 (0.5%)	1546 (0.5%)	1018 (0.5%)	
Day of injury					<0.001
Weekday (Monday–Friday)	234,439 (60.3%)	200,146 (61.2%)	184,399 (62.6%)	142,404 (67.2%)	
Weekend (Saturday–Sunday)	154,135 (39.7%)	126,586 (38.7%)	110,053 (37.4%)	69,373 (32.7%)	
Unknown	48 (0.0%)	58 (0.0%)	45 (0.0%)	60 (0.0%)	
Time of injury					<0.001
Day (07:00–14:59)	93,865 (24.2%)	96,117 (29.4%)	102,551 (34.8%)	91,174 (43.0%)	
Evening (15:00–22:59)	164,829 (42.4%)	146,090 (44.7%)	135,873 (46.1%)	86,003 (40.6%)	
Night (23:00–06:59)	129,165 (33.2%)	83,893 (25.7%)	55,286 (18.8%)	33,536 (15.8%)	
Unknown	763 (0.2%)	690 (0.2%)	787 (0.3%)	1124 (0.5%)	
Day of ER visit					<0.001
Weekday (Monday–Friday)	232,283 (59.8%)	198,260 (60.7%)	184,682 (62.7%)	145,102 (68.5%)	
Weekend (Saturday–Sunday)	156,339 (40.2%)	128,530 (39.3%)	109,815 (37.3%)	66,735 (31.5%)	
Time of ER visit					<0.001
Day (07:00–14:59)	95,699 (24.6%)	92,186 (28.2%)	95,775 (32.5%)	92,613 (43.7%)	
Evening (15:00–22:59)	166,203 (42.8%)	146,196 (44.7%)	139,766 (47.5%)	95,426 (45.0%)	
Night (23:00–06:59)	126,717 (32.6%)	88,403 (27.1%)	58,953 (20.0%)	23,795 (11.2%)	
Unknown	3 (0.0%)	5 (0.0%)	3 (0.0%)	3 (0.0%)	
Place of occurrence					<0.001
Transport area	135,451 (34.9%)	102,758 (31.4%)	98,150 (33.3%)	68,724 (32.4%)	
Home	99,432 (25.6%)	98,137 (30.0%)	87,062 (29.6%)	94,762 (44.7%)	
Commercial, recreational, or cultural area, or public building	67,353 (17.3%)	49,669 (15.2%)	36,261 (12.3%)	14,225 (6.7%)	
Sport and athletics area	26,115 (6.7%)	13,746 (4.2%)	4954 (1.7%)	1469 (0.7%)	
Farm, industrial or construction area	19,894 (5.1%)	34,490 (10.6%)	36,023 (12.2%)	9222 (4.4%)	
Countryside	12,303 (3.2%)	15,450 (4.7%)	20,425 (6.9%)	10,514 (5.0%)	
Medical service area	9451 (2.4%)	3683 (1.1%)	4112 (1.4%)	6161 (2.9%)	
School/educational area	7327 (1.9%)	1228 (0.4%)	715 (0.2%)	263 (0.1%)	
Residential institution	5317 (1.4%)	2548 (0.8%)	2595 (0.9%)	4245 (2.0%)	
Others and unknown	5979 (1.5%)	5081 (1.6%)	4200 (1.4%)	2252 (1.1%)	
Insurance					<0.001
National health insurance	294,487 (75.8%)	248,121 (75.9%)	219,986 (74.7%)	165,468 (78.1%)	
Vehicle	62,142 (16.0%)	48,080 (14.7%)	43,387 (14.7%)	26,704 (12.6%)	
Self-pay (uninsured)	22,612 (5.8%)	18,402 (5.6%)	15,870 (5.4%)	5996 (2.8%)	
Medical care beneficiary	6646 (1.7%)	9706 (3.0%)	12,974 (4.4%)	13,050 (6.2%)	
Others and unknown	2735 (0.7%)	2481 (0.8%)	2280 (0.8%)	619 (0.3%)	
Alcohol use					<0.001
No evidence of alcohol use	285,737 (73.5%)	247,543 (75.7%)	231,356 (78.6%)	181,694 (85.8%)	
Alcohol use by the injured person	49,981 (12.9%)	44,712 (13.7%)	35,497 (12.1%)	11,961 (5.6%)	
No information available	36,683 (9.4%)	23,097 (7.1%)	20,679 (7.0%)	17,057 (8.1%)	
Alcohol use by both the injured person and other person(s) involved	14,045 (3.6%)	9541 (2.9%)	5516 (1.9%)	668 (0.3%)	
Alcohol use by other person(s) involved	2176 (0.6%)	1897 (0.6%)	1449 (0.5%)	457 (0.2%)	
Intent					<0.001
Unintentional	339,453 (87.3%)	288,289 (88.2%)	269,673 (91.6%)	201,006 (94.9%)	
Assault	33,235 (8.6%)	24,071 (7.4%)	15,081 (5.1%)	3028 (1.4%)	
Intentional self-harm	14,286 (3.7%)	12,764 (3.9%)	8211 (2.8%)	6514 (3.1%)	
Others and unknown	1648 (0.4%)	1666 (0.5%)	1532 (0.5%)	1289 (0.6%)	

EMS: Emergency medical service.

**Table 2:** Mechanism and activity when injured of injury patients by age group.

Age Group (Years)	Group 1 (18–34) (*n* = 388,622)	Group 2 (35–49) (*n* = 326,790)	Group 3 (50–64) (*n* = 294,497)	Group 4 (≥65 Years) (*n* = 211,837)	*p* Value
Mechanism of injury					<0.001
Blunt force	94,262 (24.3%)	71,339 (21.8%)	49,492 (16.8%)	17,647 (8.3%)	
Transport injury event	86,920 (22.4%)	65,336 (20.0%)	61,727 (21.0%)	40,836 (19.3%)	
Falling, slipping	72,918 (18.8%)	71,001 (21.7%)	88,299 (30.0%)	112,310 (53.0%)	
Piercing/penetrating force	60,011 (15.4%)	47,350 (14.5%)	35,402 (12.0%)	11,908 (5.6%)	
Physical-over-exertion	20,346 (5.2%)	14,427 (4.4%)	8639 (2.9%)	5295 (2.5%)	
Exposure to chemical or other substance	12,223 (3.1%)	13,133 (4.0%)	11,031 (3.7%)	8929 (4.2%)	
Thermal mechanism	9948 (2.6%)	7609 (2.3%)	5553 (1.9%)	1843 (0.9%)	
Other mechanical force	2764 (0.7%)	4544 (1.4%)	5121 (1.7%)	1292 (0.6%)	
Threat to breathing	1244 (0.3%)	1450 (0.4%)	1307 (0.4%)	1240 (0.6%)	
Exposure to natural disaster or other force of nature	21 (0.0%)	18 (0.0%)	26 (0.0%)	18 (0.0%)	
Others and unknown	27,965 (7.2%)	30,583 (9.4%)	27,900 (9.5%)	10,519 (5.0%)	
Activity when injured					<0.001
Vital activity	128,651 (33.1%)	110,447 (33.8%)	106,201 (36.1%)	112,136 (52.9%)	
Leisure, play or travel	83,780 (21.6%)	59,270 (18.1%)	54,849 (18.6%)	34,144 (16.1%)	
Paid work	49,372 (12.7%)	55,484 (17.0%)	54,781 (18.6%)	14,716 (6.9%)	
Unpaid work	44,040 (11.3%)	44,598 (13.6%)	41,893 (14.2%)	32,322 (15.3%)	
Sports and exercise during leisure time	26,191 (6.7%)	14,595 (4.5%)	7373 (2.5%)	2564 (1.2%)	
Others and unknown	56,588 (14.6%)	42,396 (13.0%)	29,400 (10.0%)	15,955 (7.5%)	

**Table 3:** Number of adults who wore a safety seat belt and cars in which airbags were installed at the time of the transport injury that occurred in a car, by age group, in 2016–2017.

Age Group (Years)	Group 1 (18–34)	Group 2 (35–49)	Group 3 (50–64)	Group 4 (≥65 Years)	*p* Value
Safety seat belt	(*n* = 13,705)	(*n* = 12,054)	(*n* = 10,895)	(*n* = 5016)	<0.001
Yes	9295 (67.8%)	9038 (75.0%)	7725 (70.9%)	2965 (59.1%)	
No	3648 (26.6%)	2270 (18.8%)	2409 (22.1%)	1658 (33.1%)	
Unknown	762 (5.6%)	746 (6.2%)	761 (7.0%)	393 (7.8%)	
Air bag installation	(*n* = 4125)	(*n* = 3739)	(*n* = 2976)	(*n* = 1156)	<0.001
Yes	4125 (30.1%)	3739 (31.0%)	2976 (27.3%)	1156 (23.0%)	
No	1747 (12.7%)	1436 (11.9%)	1599 (14.7%)	977 (19.5%)	
Unknown	7833 (57.2%)	6879 (57.1%)	6320 (58.0%)	2883 (57.5%)	

**Table 4:** Clinical outcomes of injury patients in the emergency room by age group.

Age Group (Years)	Group 1 (18–34) (*n* = 388,622)	Group 2 (35–49) (*n* = 326,790)	Group 3 (50–64) (*n* = 294,497)	Group 4 (≥5 Years) (*n* = 211,837)	*p* Value
ED disposition					<0.001
Discharge	333,501 (85.8%)	263,123 (80.5%)	215,058 (73.0%)	120,926 (57.1%)	
Admission to ward	27,103 (7.0%)	30,880 (9.4%)	41,296 (14.0%)	53,780 (25.4%)	
Against medical advice	9955 (2.6%)	10,314 (3.2%)	8831 (3.0%)	5449 (2.6%)	
Transfer	9174 (2.4%)	10,398 (3.2%)	12,699 (4.3%)	13,770 (6.5%)	
Admission to ICU	6911 (1.8%)	9378 (2.9%)	13,573 (4.6%)	14,604 (6.9%)	
Death in ED	1168 (0.3%)	1643 (0.5%)	2236 (0.8%)	3106 (1.5%)	
Others and unknown	810 (0.2%)	1054 (0.3%)	804 (0.3%)	202 (0.1%)	
Operation					<0.001
No	247,324 (63.6%)	207,155 (63.4%)	180,709 (61.4%)	119,132 (56.2%)	
Yes	12,691 (3.3%)	14,972 (4.6%)	19,178 (6.5%)	23,747 (11.2%)	
Unknown	128,607 (33.1%)	104,663 (32.0%)	94,610 (32.1%)	68,958 (32.6%)	
EMR-ISS					<0.001
Mild (EMR-ISS 1–8)	211,228 (54.4%)	168,032 (51.4%)	134,069 (45.5%)	61,275 (28.9%)	
Moderate (EMR-ISS 9–24)	146,664 (37.7%)	125,210 (38.3%)	120,290 (40.8%)	107,967 (51.0%)	
Severe (EMR-ISS 25–74)	24,832 (6.4%)	27,201 (8.3%)	32,337 (11.0%)	32,711 (15.4%)	
Critical (EMR-ISS ≥ 75 or death)	2382 (0.6%)	3406 (1.0%)	5199 (1.8%)	7702 (3.6%)	
ED stay time, median (IQR), hours	1.7 (0.9–3.2)	1.9 (1.0–3.6)	2.2 (1.2–4.3)	3.2 (1.7–6.1)	<0.001
Hospital death					<0.001
No	386,900 (99.6%)	324,191 (99.2%)	290,570 (98.7%)	205,236 (96.9%)	
Yes	1722 (0.4%)	2599 (0.8%)	3927 (1.3%)	6601 (3.1%)	
Traumatic brain injury (TBI)					<0.001
No	356,029 (91.6%)	296,478 (90.7%)	257,528 (87.4%)	177,114 (83.6%)	
Yes	32,593 (8.4%)	30,312 (9.3%)	36,969 (12.6%)	34,723 (16.4%)	
Multiple injuries in anatomical sites					<0.001
No	342,091 (88.0%)	286,697 (87.7%)	253,025 (85.9%)	179,248 (84.6%)	
Yes	46,531 (12.0%)	40,093 (12.3%)	41,472 (14.1%)	32,589 (15.4%)	

ED: Emergency department; ICU: Intensive care unit; EMR-ISS: Excess mortality ratio-based injury severity score.

**Table 5:** Logistic regression analysis on clinical outcomes of injury patients (ERM-ISS, Hospital Death, and TBI).

Variable	EMR-ISS (Severe, Critical)				Hospital Death				TBI			
	Univariable Analysis		Multivariable Analysis		Univariable Analysis		Multivariable Analysis		Univariable Analysis		Multivariable Analysis	
	OR	95% CI	AOR	95% CI	OR	95% CI	AOR	95% CI	OR	95% CI	AOR	95% CI
Age group (years)												
(Ref) Group 1 (18–34)	1.00		1.00		1.00		1.00		1.00		1.00	
Group 2 (35–49)	1.37	1.35–1.4	1.19	1.17–1.22	1.37	1.35–1.4	1.56	1.46–1.66	1.12	1.10–1.14	1.11	1.09–1.13
Group 3 (50–64)	1.94	1.91–1.97	1.61	1.58–1.64	1.94	1.91–1.97	2.40	2.25–2.55	1.57	1.54–1.59	1.42	1.39–1.44
Group 4 (≥65 years)	3.13	3.08–3.18	2.29	2.24–2.34	3.13	3.08–3.18	4.68	4.40–4.97	2.14	2.11–2.18	1.60	1.57–1.63

ESM: Emergency medical service; EMR-ISS: Excess mortality ratio-based injury severity score; TBI: Traumatic brain injury; OR: Odds ratio; CI: Confidence interval; AOR: Adjusted odds ratio.

## Data Availability

The data that support the findings of this study are available from the Korea Center for Disease Control (KCDC). Restrictions apply to the availability of these data, which were used under license for this study. Data are available with the permission of KCDC.

## Supplementary Materials

The following are available online at https://www.mdpi.com/1660-4601/18/3/1210/s1, Table S1. Equivalents of ICECI version 1.2 and EDIIS codes: Place of occurrence, mechanism of injury, activity when injured, alcohol use, intent.
