# Supplementary material for: National Surveillance of Injury in the Republic of Korea: Increased Injury Vulnerability in the Late Middle Age
Source: Int J Environ Res Public Health. 2021 Jan 29;18(3):1210. doi: 10.3390/ijerph18031210 (PMC7908217; doi:10.3390/ijerph18031210)
Supplement: Supplementary file 1 [file ijerph-18-01210-s001.pdf]

Table S1 Equivalents of ICECI version 1.2 and EDIIS codes: Place of occurrence, Mechanism of injury, Activity when injured, Alcohol use, Intent.

manuscript	ICECI		EDIIS
**Place of occurrence**	**Place of occurrence (C4=1-12, 98, 99)**		**Place of occurrence (23)**
- Transport area	- Transport area: public highway, street or road (C4=6)		C6(C6.1, C6.2, C6.3, C6.8, C6.9)
	- Transport area: other (C4=7)		C7(C7.1,C7.3, C7.9,C7.9)
- Home	- Home (C4=1)		C1
- Commercial, recreational, cultural area, or public building	- Commercial area (11)		C11
	- Recreational area, cultural area or public building (C4=10)		C10
- Sport and athletics area	- Sport and athletics area (C4=5)		C5(C5.1,C5.2,C5.3,C5.6,C5.7,C5.8,C5.9)
- Farm, industrial or construction area	- Farm or other place of primary production (C4=9)		C9
	- Industrial or construction area (C4=8)		C8
- Countryside	- Countryside (C4=12)		C12
- Medical service area	- Medical service area (C4=3)		C3
- School/educational area	- School, educational area (C4=4)		C4
- Residential institution	- Residential institution (C4=2)		C2
- Others and unknown	- Other specified place of occurrence (C4=98)		C98
	- Unspecified place of occurrence (C4=99)		C99
**Mechanism of injury**	**Mechanism of injury (C2=1-8, 20, 98, 99)**		**Mechanism of injury (20)**
- Transport injury event	- Blunt force (C2=1) (C2=1.1, 1.2, 1.3, 1.4, 1.5, 1.6, 1.8, 1.9)	- Transport injury event (C2=1.1)	C11 (C11.10, C11.20, C11.45, C11.61, C11.67, C11.70, C11.82, C11.84, C11.88, C11.90, C11.92, C11.98, C11.99)
- Falling, slipping		- Falling, stumbling, jumping pushed (C2=1.5)	C12 (C12.0, C12.1, C12.2, C12.3, C12.4, C12.5, C12.6, C12.7, C12.8, C12.9)
- Blunt force		- Contact with object or animal (C2=1.2),<br>- Contact with person (C2=1.3),<br>- Crushing (C2=1.4),<br>- Abrading, rubbing (C2=1.6),<br>- Other specified contact with blunt force (C2=1.8),<br>- Unspecified contact with blunt force (C2=1.9)	C13 (C13.2 (C13.21, C13.22, C13.23, C13.25, C13.28, C13.29)<br>C13.3, C13.4, C13.9)
- Piercing/penetrating force	- Piercing/penetrating force (C2=2)		C2 (C2.1, C2.2 (C2.20, C2.22, C2.23, C2.24), C2.4 (C2.41, C2.42, C2.43, C2.45, C2.47, C2.48), C2.8, C2.9)
- Other mechanical force	- Other mechanical force (C2=3)		C3(C3.1, C3.2, C3.8, C3.9).
- Physical-over-exertion	- Physical-over-exertion (C2=7)		C7

- Exposure to chemical or other substance	- Exposure to chemical or other substance (C2=6)	C6
- Thermal mechanism	- Thermal mechanism (C2=4)	C4 (C4.1(C4.11, C4.12, C4.13, C4.14, C4.15, C4.16, C4.17, C4.18, C4.19), C4.2 (C4.21, C4.22, C4.28. C4.29), C4.8, C4.9)
- Threat to breathing	- Threat to breathing (C2=5)	C5.2 (C5.21, C5.22, C5.23, C5.24, C5.25), C5.3, C5.8, C5.9
- Exposure to natural disaster or other force of nature	- Exposure (effect of) to whether, natural disaster, or other force of nature (C2=8)	C8 (C8.1, C8.2, C8.5, C8.8, C8.9)
- Others and unknown	- Complications of health care (C2=20)	
	- Other specified mechanism of injury (C2=98)	C98 (C98.1 (C98.11, C98.12, C98.13, C98.14, C98.15, C98.18, C98.19) C98.2 (C98.21, C98.22, C98.24, C98.27), C98.3, C98.4, C98.6), C98.8
	- Unspecified mechanism of injury (C2=99)	C99
**Activity when injured**	**Activity when injured (C5=1-8, 98, 99)**	**Activity when injured (27)**
- Vital activity	- Vital activity (C5=6)	C5
- Leisure, play or travel	- Leisure or play (C5=5)	C6
	- Travelling not elsewhere classified (C5=8)	C8
- Paid work	- Paid work (C5=1)	C1
- Unpaid work	- Unpaid work (C5=2)	C2
- Sports and exercise during leisure time	- Sports and exercise during leisure time (C5=4)	C4
- Others and unknown	- Education (C5=3)	C3
	- Being taken care of (C5=7)	C7
	- Other specified activity (C5=98)	C98
	- Unspecified activity (C5=99)	C99
**Alcohol use**	**Alcohol use (C6=1-5)**	**Alcohol use (19)**
- No evidence of alcohol use	- No suspicion or evidence of alcohol use by any person involved in the injury event (C6=2)	C2
- Alcohol use by the injured person	- Suspicion or evidence of alcohol use by the injured person (C6=3)	C3
- No information available	- No information available (C6=1)	C1
- Alcohol use by the both injured person and other person/s involved	- Suspicion or evidence of alcohol use by both the injured person and other persons involved in the injury event (C6=5)	C5
- Alcohol use by other person/s involved	- Suspicion or evidence of alcohol use by other persons involved in the injury event (C6=4)	C4
**Intent**	**Intent (C1=1-6, 8, 9)**	**Intent (18)**
- Unintentional	- Unintentional (C1=1)	C1
- Assault	- Assault (C1=3)	C3

- Intentional self-harm	- Intentional self-harm (C1=2)	C2
- Others and unknown	- Other Violence (C1=4)	C98
	- Undetermined intent (C1=5)	
	- Complications of medical or surgical care (C1=6)	
	- Other specified intent (C1=8)	
	- Unspecified intent (C1=9)	
